# Ticagrelor versus clopidogrel after intracranial stent angioplasty: a real-world study

**DOI:** 10.3389/fneur.2023.1232958

**Published:** 2023-08-11

**Authors:** Lili Sun, Shuzhen Wang, Yun Song, Wei Zhao, Meimei Zheng, Hao Yin, Jun Zhang, Yao Meng, Wei Wang, Ju Han

**Affiliations:** ^1^Shandong Key Laboratory of Rheumatic Disease and Translational Medicine, Department of Neurology, Shandong Institute of Neuroimmunology, The First Affiliated Hospital of Shandong First Medical University and Shandong Provincial Qianfoshan Hospital, Jinan, China; ^2^Shandong Engineering and Technology Research Center for Pediatric Drug Development, Department of Pediatrics, The First Affiliated Hospital of Shandong First Medical University and Shandong Provincial Qianfoshan Hospital, Jinan, China

**Keywords:** ticagrelor, clopidogrel, antiplatelet therapy, intracranial stent, ischemic stroke

## Abstract

**Objective:**

It was unknown whether a regimen of aspirin plus ticagrelor (aspirin-ticagrelor) attenuates ischemic vascular events without increasing bleeding risk in patients who had undergone intracranial stenting compared with an aspirin plus clopidogrel (aspirin-clopidogrel) regimen. This article compares the efficacy and safety outcomes of the two double antibody regimens in patients undergoing intracranial stent and investigates whether aspirin-ticagrelor could be an alternative antiplatelet agent without increasing the risk of bleeding.

**Methods:**

We conducted a retrospective analysis of our database for patients who had undergone intracranial stenting. From January 2017 to May 2021, consecutive patients treated with endovascular stenting were identified and dichotomized by whether aspirin-ticagrelor or aspirin-clopidogrel were used. The outcomes were compared by propensity score matching.

**Results:**

A total of 340 patients treated with intracranial stent were included. Of all, 132 patients were matched. At 180 days, ischemic vascular events occurred in one patient (1.5%) in the aspirin-ticagrelor group and in six patients (9.1%) in the aspirin-clopidogrel group. Although the absolute incidence of ischemic vascular events [1.5% (1/66) vs. 9.1% (6/66), *p* = 0.125] was lower in the aspirin-ticagrelor group than in the aspirin-clopidogrel group, there were no statistical differences. There were no statistical differences in ischemic vascular events, ischemic stroke, or death up to 180 days between the two groups. In addition, the incidence of bleeding did not differ. No intracranial hemorrhage or mild bleeding occurred. No statistically significant difference was noted in restenosis and symptomatic restenosis at follow-up.

**Conclusion:**

In our study involving patients with acute ischemic stroke who had undergone intracranial stenting, aspirin-ticagrelor was not found to be superior to aspirin-clopidogrel in reducing the rate of ischemic vascular events. The risk of bleeding did not differ between the two groups. Aspirin-ticagrelor does not lower total restenosis and symptomatic restenosis risk at follow-up.

## Introduction

Antiplatelet therapy is recommended after non-cardiogenic ischemic stroke, aiming at limiting thrombosis on ulcer atherosclerotic plaque and subsequent distal embolism (1). Dual antiplatelet therapy (DAPT) with a P2Y12 receptor inhibitor is often recommended after neuroendovascular stenting, with a transition to aspirin monotherapy after follow-up endovascular angiography (2, 3). To maintain the trade-off between ischemic and bleeding risk, neuro interventionists tried to apply an escalation individualized antiplatelet treatment strategy by replacing clopidogrel with ticagrelor in specific patients (4–6). Clopidogrel needs to be converted into its active form through the liver, which is ineffective in 25% of white patients and 60% of Asian patients, so the efficacy in these patients is uncertain (7, 8).

Ticagrelor is a reversible antagonist, which can directly block the P2Y12 receptor of platelets. Its antiplatelet effect does not need metabolic activation, and its level of inhibition of platelet aggregation may be higher than that of clopidogrel (9, 10). Ticagrelor has been approved for the treatment of acute coronary syndrome (11, 12) and is presumably to be used for transient ischemic attack (TIA) and mild stroke. Aspirin plus ticagrelor (aspirin-ticagrelor) was superior to aspirin in reducing death or stroke among patients with TIA or acute mild-to-moderate ischemic stroke (13). In the PRINCE trial, patients with TIA or minor stroke who were treated with aspirin-ticagrelor had a lower proportion of high platelet reactivity than those who were treated with aspirin plus clopidogrel (aspirin-clopidogrel) (14). The recent CHANCE-2 trial in China showed that among patients with TIA or minor ischemic stroke who were carriers of CYP2C19 loss-of-function alleles, the risk of stroke at 90 days in the ticagrelor group was modestly lower than that in the clopidogrel group. There was no difference in the risk of moderate or severe bleeding between the two treatment groups, but ticagrelor was associated with more total bleeding events than clopidogrel (15).

These trials have not yet reported results for patients with intracranial stenosis who had undergone stenting. Further clinical studies are warranted to directly compare aspirin-ticagrelor with aspirin-clopidogrel in patients with an intracranial stent. Thus, our study aimed to investigate the safety and efficacy of aspirin-ticagrelor versus aspirin-clopidogrel in reducing ischemic vascular events and death in patients with DAPT after intracranial stenting.

## Methods

### Patients

**Figure 1:**
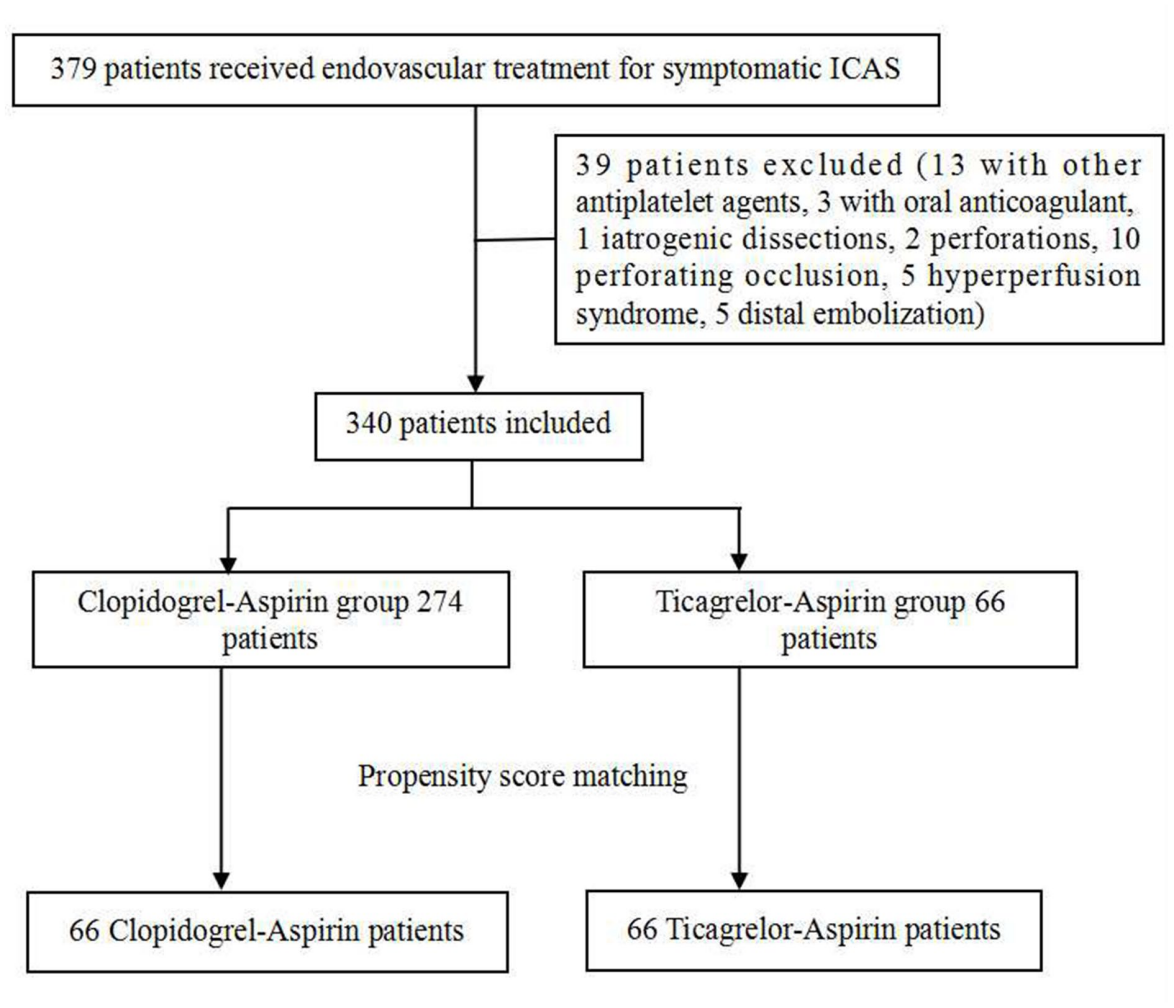
Patient flowchart. Flowchart of the study. ICAS, intracranial atherosclerotic stenosis.

We retrospectively reviewed our cerebrovascular disease database to identify patients who had been treated with an endovascular stent for symptomatic, severe (stenosis degree 70% to 99%) intracranial atherosclerotic stenosis (ICAS) between January 2017 and May 2021. All the patients had recurrent strokes after aggressive medical management, so stent angioplasty was advised. Only patients on aspirin-ticagrelor or aspirin-clopidogrel after stenting were included, which resulted in the exclusion of patients treated with clopidogrel or aspirin alone and those treated with other antiplatelet drugs or anticoagulants. Patients with procedure-related adverse events, such as perforation or dissections, and distal embolization were excluded. The excluded patients are listed in Figure 1. The patients who had undergone stenting and received DAPT with aspirin-ticagrelor were compared with those who received aspirin-clopidogrel.

The study protocol was approved by our institutional review board and all patients gave informed consent before the operation.

### Perioperative management and intervention procedure

All patients were on DAPT daily for at least 5 days before stenting. Thrombelastogram was performed to guide antiplatelet therapy. If used, ticagrelor was given with a dose load of 180 mg, followed by 90 mg twice daily. The interventional procedures were similar to those reported in a previous trial (16). Patients were required to take aspirin 100 mg/day and clopidogrel 75 mg/day or ticagrelor 90 mg twice daily for 180 days and then continued with either one of the two antiplatelet drugs. They were also prescribed statins and educated on how to control other risk factors. Non-enhanced computed tomographic (CT) scans were performed on all patients immediately after the procedure of endovascular intervention.

### Data collection and follow-up outcomes

Patients were systematically followed up by trained followers 1 and 6 months after vascular intervention. They were scheduled to return for a vascular imaging examination at 6 months after the endovascular intervention. All the follow-up times included in the analysis were censored at 180 days if longer. If patients showed any signs of neurological deterioration during the follow-up period after the intervention, appropriate imaging, such as magnetic resonance imaging (MRI) or CT, was used to confirm the existence of hemorrhagic or ischemic complications. The following information was collected: demographic data, clinical outcomes, medication compliance, changes in antiplatelet agents, and adverse drug reactions. The information was collected from electronic medical records at every admission for follow-up, telephone interviews, or clinic visits. The primary efficacy outcome was ischemic vascular events, which was a composite of ischemic stroke, transient ischemic attacks (TIA), myocardial infarction, or death from vascular causes. Ischemic strokes were classified as minor or major strokes. TIA, a minor stroke, and a major stroke were determined according to the measurements of acute cerebral infarction recommended by Brott et al. (17). In addition, ischemic stroke was further classified according to the etiology into cerebral embolism, perforating artery occlusive cerebral infarction, hypoperfusion type, and unclassifiable type. Owing to the retrospective design of the study, it was not possible to distinguish the exact etiology of all ischemic strokes. The secondary efficacy outcomes were disability based on the modified Rankin scale (mRS), with scores ranging from 0 to 6 (no symptoms to death), at the end of the follow-up visit 180 days after the intervention (18). Specifically, the secondary clinical efficacy outcomes included the proportion of patients with an mRS score of 0 to1, or who returned to their premorbid mRS score, the proportion of patients with an mRS score of 0 to 2, and the proportion of patients with an mRS score of 0 to 3 at 180 days. The angiographic follow-up outcomes were angiographic restenosis and symptomatic restenosis at 180 days. Restenosis was defined as stenosis >50% in the stent or immediately adjacent (within 5 mm) of the stent and > 20% absolute luminal loss (19). Restenosis associated with ischemic symptoms of the offending vessel territory was defined as symptomatic restenosis. The primary safety outcome was moderate or severe bleeding as defined by the Global Utilization of Streptokinase and Tissue Plasminogen Activator for Occluded Coronary Arteries (GUSTO) criteria at 180 days (20). Intracranial hemorrhage (ICH) was classified according to the Heidelberg hemorrhage scale (HBC) (21). The secondary safety outcomes included any bleeding, mild bleeding, and other adverse events through 180 days of follow-up.

### Statistical analysis

Categorical variables were compared using the chi-square or Fisher’s exact tests; continuous variables were analyzed by student *t*-tests for normally distributed data or Mann–Whitney *U* tests for skewed data. Propensity score matching (PSM) was performed to compare the outcomes. The Wilcoxon signed-rank test for numerical variables and McNemar’s test for categorical variables were used to test the differences in baseline characteristics and outcomes after 1:1 matching with a caliper width of 0.02 of the propensity score. All analyses were performed using SPSS version 24.0 (IBM, Armonk, New York).

## Results

### General subject characteristics

A total of 379 patients treated with endovascular stent for symptomatic severe ICAS were screened between January 2017 and May 2021. A total of 39 patients were excluded. Finally, 340 patients were included in the study, with 274 in the aspirin-clopidogrel group and 66 in the aspirin-ticagrelor group. Of the 340 patients, none of them had more than 40% of postoperative stenosis. A total of 132 patients were matched after PSM, with 66 patients in each group. Figure 1 shows the patient flowchart. To better keep the consistency, only patients with digital subtraction angiography or computer tomography angiography (DSA/CTA) outcomes were included in the angiographic follow-up outcomes of this study. Overall, 80.3% of the patients had complete follow-up for the DSA/CTA outcomes of the PSM patients [83.3% (55/66) vs. 77.3% (51/66)].

### Baseline characteristics before and after propensity score matching

The characteristics of the patients at baseline were similar in the two treatment groups (Table 1). The median age of the patients was 60.18 years, and 70.6% were men.

**Table 1:** Baseline characteristics of the patients before propensity score matching (PSM).

Characteristic	Clopidogrel-aspirin group (*n* = 274)	Ticagrelor-aspirin group (*n* = 66)	*p*-value
*Demographics*			
Age, mean ± SD, years	59.9 ± 9.5	60.3 ± 9.7	0.745
Male, *n* (%)	203 (74.1)	37 (56.1)	0.004
*Medical history, n (%)*			
Hypertension	196 (71.5)	45 (68.2)	0.591
Diabetes mellitus	120 (43.8)	22 (33.3)	0.122
Coronary artery disease	24 (8.8)	7 (10.6)	0.64
Smoking, *n* (%)	118 (43.1)	21 (31.8)	0.095
Family history, *n* (%)	44 (16.1)	14 (21.2)	0.318
*Stenosis location, n (%)*			
Anterior circulation	134 (48.9)	33 (50.0)	0.873
Posterior circulation	140 (51.1)	33 (50.0)	
Admission NIHSS score, median (IQR)	1 (0–3)	2 (0–4)	0.337

Before PSM, there were no significant differences in baseline characteristics between the two groups, except for a significantly higher rate of male patients in the aspirin-clopidogrel group (Table 1). After PSM, the baseline parameter was well-balanced between the two groups (Table 2).

**Table 2:** Baseline characteristics of the matched patients.

Characteristic	Clopidogrel-aspirin group (*n* = 66)	Ticagrelor-aspirin group (*n* = 66)	*p*-value
*Demographics*			
Age, mean ± SD, years	59.6 ± 9.3	61.6 ± 8.3	0.9
Male, *n* (%)	45 (68.2)	37 (56.1)	0.229
*Medical history, n (%)*			
Hypertension	50 (75.8)	45 (68.2)	0.424
Diabetes mellitus	27 (40.9)	22 (33.3)	0.473
Coronary artery disease	7 (10.6)	7 (10.6)	1
Smoking, *n* (%)	31 (47.0)	21 (31.8)	0.143
Family history, *n* (%)	11 (16.7)	14 (21.2)	0.678
*Stenosis location, n (%)*			
Anterior circulation	39 (59.1)	33 (50.0)	0.362
Posterior circulation	27 (40.9)	33 (50.0)	
Admission NIHSS score, median (IQR)	2 (0–3.3)	2 (0–4)	0.675

### The outcome of the patients after propensity score matching

#### Efficacy outcomes

The primary-outcome event, ischemic vascular events within 180 days, occurred in 1 of the 66 patients (1.5%) in the aspirin-ticagrelor group and in 6 of the 66 patients (9.1%) in the aspirin-clopidogrel group (Table 3). However, although the absolute incidence of recurrent ischemic events was lower in the aspirin-ticagrelor group than those in the aspirin-clopidogrel group, there were no statistical differences [1.5% (1/66) vs. 9.1% (6/66), *p* = 0.125] (Table 3).

**Table 3:** Efficacy and safety endpoints of the matched patients.

Characteristic	Clopidogrel-aspirin group (*n* = 66)	Ticagrelor-aspirin group (*n* = 66)	*p*-value
*Primary efficacy outcome, n (%)*			
Ischemic vascular events	6 (9.1)	1 (1.5)	0.125
Ischemic stroke	5 (7.6)	1 (1.5)	0.219
TIA	1 (1.5)	0 (0)	NA
Myocardial infarction	0 (0)	0 (0)	NA
Death	1 (1.5)	0 (0)	NA
*Secondary efficacy outcomes, n (%)*			
mRS scores at 180 days			
0 to 1	50 (75.8)	55 (83.3)	0.383
0 to 2	57 (86.4)	58 (87.9)	1
0 to 3	62 (93.9)	63 (95.5)	1
*Safety outcomes*			
Severe or moderate bleeding	1 (1.5)	1 (1.5)	1
Mild bleeding	0 (0)	0 (0)	NA
ICH	0 (0)	0 (0)	NA
Any bleeding	1 (1.5)	1 (1.5)	1
Other adverse events	0 (0)	1 (1.5)	NA

There were no statistical differences in the composite of recurrent ischemic stroke or death up to 180 days between the two groups. All of the ischemic vascular events were ischemic strokes (four minor strokes and two major strokes), except one TIA in the aspirin-clopidogrel group. According to the etiology of ischemic stroke, four patients had perforating artery occlusion, one had a hypoperfusion cerebral infarction due to gastrointestinal bleeding, and one patient had a fatal major stroke in the non-stent vascular watershed. There were no myocardial infarctions in the two groups and no death in the aspirin-ticagrelor group within 180 days.

**Figure 2:**
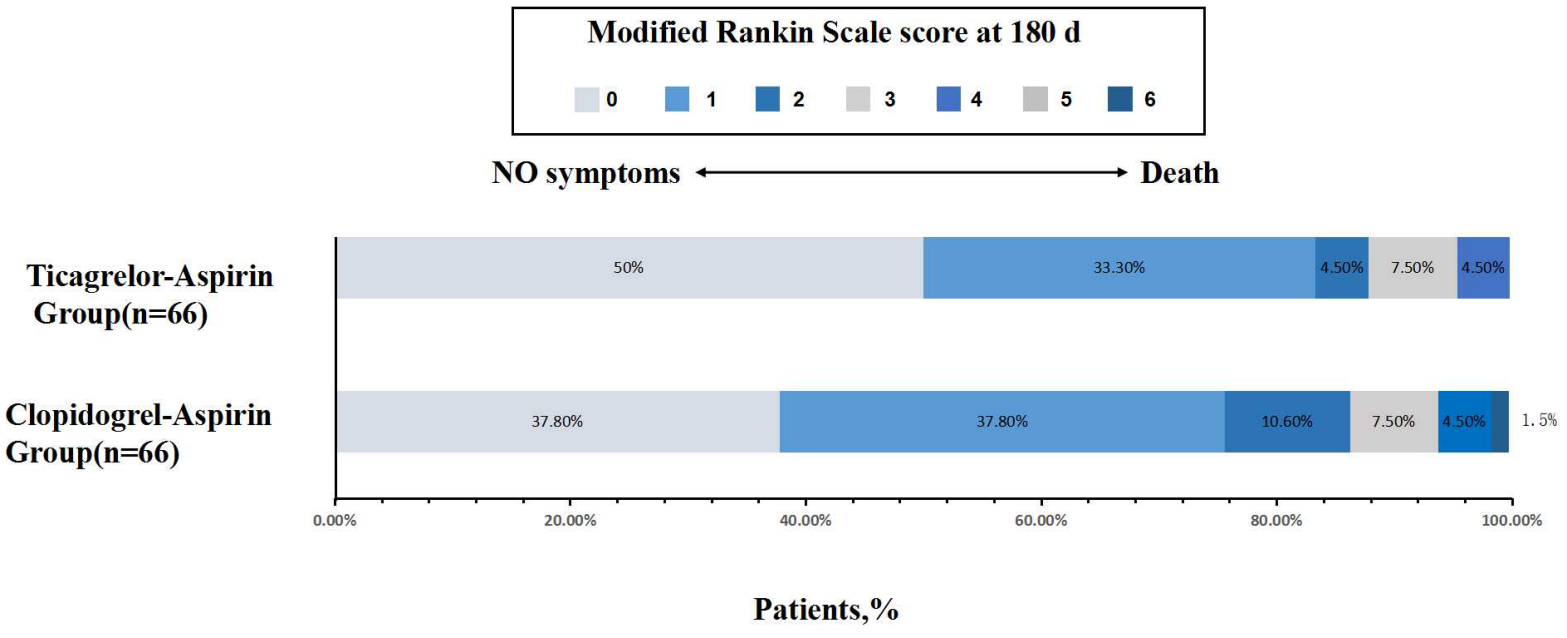
Distribution of global disability at 180 days based on the modified Rankin scale score.

**Figure 3:**
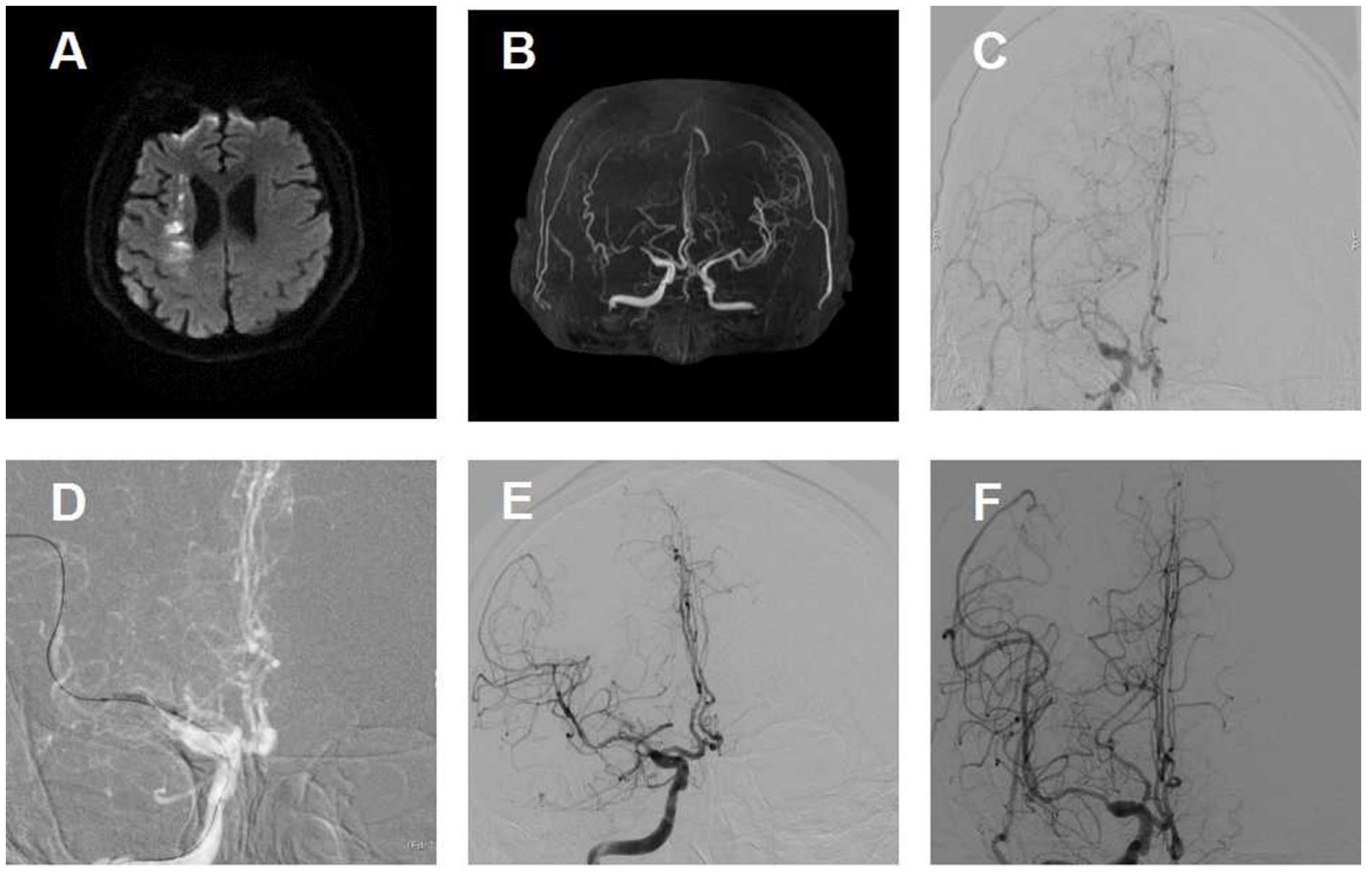
Image of a patient with severe stenosis of the middle cerebral artery (MCA) and follow-up. **(A)** Diffusion-weighted image shows an infarction in the internal border zone area of the right corona radiata. **(B)** Magnetic resonance angiographic shows right MCA severe stenosis. **(C)** Right MCA severe stenosis confirmed by digital subtraction angiography. **(D)** Predilation with a balloon. **(E)** Angiographic result after the procedure with good antegrade perfusion. **(F)** Angiographic outcome at 180 days follow-up.

No statistically significant difference was noted in all three secondary clinical outcomes. For example, the percentage of patients with an mRS score of 0 to 1 was 75.8% for the aspirin-clopidogrel group and 83.3% for the aspirin-ticagrelor group. The patients with an mRS score of 0 to 3 accounted for 93.9% of the patients in the aspirin-clopidogrel group and for 95.5% in the aspirin-ticagrelor group. The distribution of global disability at 180 days based on the mRS score is illustrated in Figure 2. There were no significant differences in baseline characteristics between patients with angiographic follow-up outcomes of the two groups. The restenosis and symptomatic restenosis outcomes at follow-up are shown in Table 4. No statistically significant difference was noted in restenosis and symptomatic restenosis at follow-up. The image and follow-up angiography of one patient with severe stenosis of the middle cerebral artery (MCA) are shown in Figure 3, and no restenosis was found.

**Table 4:** Baseline characteristics and outcomes of patients with an angiographic follow-up image.

Characteristic	Clopidogrel-aspirin group (*n* = 51)	Ticagrelor-aspirin group (*n* = 55)	*p*-value
*Demographics*			
Age, mean ± SD, years	59.5 ± 9.1	61.2 ± 9.3	0.356
Male, *n* (%)	35 (68.6)	32 (58.2)	0.265
*Medical history, n (%)*			
Hypertension	37 (72.5)	38 (69.1)	0.696
Diabetes mellitus	21 (41.2)	18 (32.7)	0.367
Coronary artery disease	6 (11.8)	7 (12.7)	0.88
Smoking, *n* (%)	22 (43.1)	16 (29.1)	0.132
Family history, *n* (%)	10 (19.6)	13 (23.6)	0.615
*Stenosis location, n (%)*			
Anterior circulation	28 (54.9)	26 (47.3)	
Posterior circulation	23 (45.1)	29 (52.7)	0.432
Admission NIHSS score, median (IQR)	2 (0–4)	2 (0–4)	0.899
Restenosis at follow-up, *n* (%)	3 (5.9)	2 (3.6)	0.931
Symptomatic restenosis at follow-up, *n* (%)	1 (2.0)	0 (0)	0.481

#### Safety outcomes

A primary safety outcome of severe or moderate bleeding, as defined by the GUSTO criteria, occurred in one patient (1.5%) in both the aspirin-ticagrelor group and the aspirin-clopidogrel group. No ICH or mild bleeding occurred in either group.

One patient in the aspirin-ticagrelor group experienced an adverse event, characterized by dyspnea and chest tightness, without discontinuation of the study treatment. No other adverse events occurred in the two groups.

## Discussion

In this single-center retrospective study, we found no statistically significant difference in efficacy between aspirin-clopidogrel and aspirin-ticagrelor in the prevention of ischemic vascular events or death in patients with intracerebral stent angioplasty after acute ischemic stroke who received 180 days treatment with DAPT. Moreover, ischemic strokes constituted most of the events in the composite primary outcome, and no difference was found between the two antiplatelet regimens in preventing their recurrence. In this single-center retrospective study, patients with intracerebral stent angioplasty after acute ischemic stroke who received treatment with aspirin-ticagrelor did not have a lower risk of ischemic vascular events at 180 days than those who received aspirin-clopidogrel, although the absolute incidence of recurrent ischemic vascular events [1.5% (1/66) vs. 9.1% (6/66), *p* = 0.125] was lower in the aspirin-ticagrelor group than in the aspirin-clopidogrel group.

For the secondary clinical efficacy outcomes, the percentage of patients with an mRS score of 0 to 1, the proportion of patients with an mRS score of 0 to 2, and the proportion of patients with an mRS score of 0 to 3 at 180 days were lower for the aspirin-ticagrelor group than for the aspirin-clopidogrel group. However, there were no statistical differences (Table 3). The incidence of total bleeding events was similar in the two treatment groups, owing to one case of moderate or severe bleeding in each group.

DAPT consisting of aspirin associated with a P2Y12 inhibitor is the mainstay of periprocedural and postprocedural treatment to reduce thromboembolic complications (2–4). In clinical practice, it is not easy to achieve a good balance between ischemia and bleeding risk in patients receiving DAPT treatment, and it is even more complicated in patients receiving an intracranial stent. Our study was designed to examine the effect of aspirin-clopidogrel as compared with aspirin-ticagrelor among patients who had undergone intracranial stenting.

Although there is strong evidence to support the use of aspirin-clopidogrel in acute mild ischemic stroke and TIA (22, 23), clopidogrel may not be a universal treatment because genetic variations may lead to the agent’s ineffectiveness (6, 24). Clopidogrel is a prodrug requiring hepatic conversion into its active metabolite by the CYP2C19 enzyme, so the process may be influenced by genetic polymorphisms (7, 8). In the literature, the prevalence of clopidogrel resistance in the general population varies greatly due to the different races and definitions used in the study population (7, 8, 25, 26). Recently, genetic testing in medicine has provided a new possibility for personalized medicine: the genetic sub-study of the CHANCE trial revealed that aspirin-clopidogrel only reduced the risk of stroke recurrence in non-carriers of the CYP2C19 loss-of-function allele (8). However, at present, it is not recommended to carry out genetic testing of clopidogrel resistance routinely. In addition, the relationship between the polymorphisms and the clinical effect of clopidogrel is controversial (27).

In functional outcome analyses of our study, the proportion of the favorable functional prognosis was higher in the aspirin-ticagrelor group than in the aspirin-clopidogrel group, although the test did not reach statistical significance. This may reflect that there is no difference between the two groups, but it may also reflect the lack of study power to evaluate the role. Randomized clinical trials have shown that aspirin-ticagrelor was associated with a lower risk of stroke at 90 days among patients with TIA or minor ischemic stroke who carried the CYP2C19 loss-of-function allele (15). The differences in patient population and outcome classification make it difficult to compare these results with those of the current study. The potential benefits of ticagrelor should be acknowledged, including its faster onset of action and more consistent platelet inhibition than clopidogrel (8, 27, 28). Almost all patients with poor response to clopidogrel will have platelet reactivity below the cutoff points related to ischemic risk when receiving ticagrelor treatment (28).

Our results support the use of aspirin-ticagrelor as an effective alternative treatment for some patients after intracranial stenting because it has a comparable effect in preventing recurrent strokes and death. Moreover, we did not find a significant increase in bleeding connected to the use of ticagrelor. Bleeding is one of the most common adverse events after endovascular intervention in large vessel stenosis stroke with DAPT. The overall rate of bleeding in this study was lower than those of previous trials involving patients with TIA or minor ischemic stroke (14). ICH is the most worrisome type of hemorrhage and predicts a bad outcome. No ICH occurred in either group of our patients. Aspirin-ticagrelor did not increase the risk of bleeding compared with aspirin-clopidogrel in patients with intracranial stent. The safety profile of ticagrelor was consistent with a previous meta-analysis study that did not show an increased risk of bleeding (29). Although the rate of adverse events was low in both groups, it seems to be more common among patients who received ticagrelor. This difference was mainly due to dyspnea, which is a known adverse effect of ticagrelor (10).

In conclusion, in our study involving patients with acute ischemic stroke who had undergone intracranial stenting, aspirin-ticagrelor was not found to be superior to aspirin-clopidogrel in reducing the risk of the composite end point of ischemic stroke, TIA, myocardial infarction, or death from vascular causes. This hypothesis still needs to be corroborated by large, prospective, randomized trials, as the matched sample size was not large enough after 1:1 PSM (66 patients in each group). In the future, in a randomized controlled trial based on the genotype detection of CYP2C19, the selection of DAPT may be more accurate and individualized. Until then, our results provide a direct comparison between the efficacy and safety of aspirin-clopidogrel vs. aspirin-ticagrelor in the intracranial stent populations in the real world.

### Study limitations

This study has some important limitations. The retrospective and observational design of the study exposes the risk of potential unmeasured confounding, which may affect the results of this study. Although the propensity score matching analysis is applied to balance the potential covariates, it cannot be matched as well as the randomized controlled trial. Second, all patients are from China, so the results may not apply to other ethnicities and races. Third, the results should be extrapolated with caution because the matched sample size was not large enough and the follow-up time was short. However, this study aimed to collect preliminary data for a future study. Future prospective randomized trials should take these variables into account in their analyses.

## Conclusion

In our study involving patients with acute ischemic stroke who had undergone intracranial stenting, aspirin-ticagrelor was not found to be superior to aspirin-clopidogrel in reducing the rate of ischemic vascular events at 180 days. The risk of bleeding did not differ between the two treatment groups. Aspirin-ticagrelor can not lower total restenosis and symptomatic restenosis risk at 180 days follow-up. Further trials on this topic could provide more information regarding the safety and efficacy of the administration of aspirin-ticagrelor in these patients.

## Data availability statement

The original contributions presented in the study are included in the article/supplementary material, further inquiries can be directed to the corresponding author.

## Ethics statement

The studies involving humans were approved by The First Affiliated Hospital of Shandong First Medical University & Shandong Provincial Qianfoshan Hospital. The studies were conducted in accordance with the local legislation and institutional requirements. Written informed consent for participation in this study was provided by the participants’ legal guardians/next of kin.

## Author contributions

LS, JH, SW, and YS: conception and design. LS, WZ, and MZ: analysis and interpretation. JZ, HY, WW, SW, and YM: data collection. LS, WZ, MZ, JZ, and YM wrote the main manuscript. LS, JZ, YS, and JH: critical revision. LS, HY, WW, and JH: statistical analysis. All authors contributed to the article and approved the submitted version.

## Conflict of interest

The authors declare that the research was conducted in the absence of any commercial or financial relationships that could be construed as a potential conflict of interest.

## Publisher’s note

All claims expressed in this article are solely those of the authors and do not necessarily represent those of their affiliated organizations, or those of the publisher, the editors and the reviewers. Any product that may be evaluated in this article, or claim that may be made by its manufacturer, is not guaranteed or endorsed by the publisher.
